# Placenta Prævia—Version—Craniotomy—Death of Mother

**Published:** 1880-05

**Authors:** 


PLACENTA PRÆVIA—VERSION—CRANIOTOMY—DEATH OF MOTHER.—M. K., aged thirty-four; sixth pregnancy. A week before admission she had a severe attack of hemorrhage, lasting about two hours; the blood was both fluid and in clots.

August 10—Hemorrhage recommenced about 6 in the morning and continued until 5:30 P.M., when she was admitted into the hospital. On examination the os was discovered as large as a florin and almost entirely covered by the placenta. This was separated from the cervical zone of the uterus, and the membranes ruptured. After this not only did the hemorrhage entirely cease, but labor also was arrested, and for three days things remained *in statu quo.*

On the 13th the discharge was most offensive, the pulse rose to 130, and temperature to 104. Barnes' bags was introduced, and the os sufficiently dilated to deliver. This was attempted by version, but after the birth of the body and arms, the head could not be brought through the brim. The forceps was tried, but failed; and the child being evidently lifeless, the head was perforated behind the ear, and crushed with the cephalotrite. There was some hemorrhage during extraction, but none afterwards; the pulse was very weak throughout. Afterwards she was given beef tea and brandy freely; morphia, injected hypodermically, gave her a quiet night's rest; pulse 115.

August 14 - Towards evening vomiting set in, but was checked by morphia, after which she slept.

August 15—Vomiting set in again; pulse rapid and weak; discharge very offensive; died at 11 P. M.—*Dublin Medical Journal.*

